# Prevalence of Postpartum Depression in a Tertiary Health Care

**DOI:** 10.31729/jnma.4805

**Published:** 2020-03-31

**Authors:** Priza Pradhananga, Prajita Mali, Lisasha Poudel, Minani Gurung

**Affiliations:** 1 Department of Public Health, Om Health Campus, Kathmandu, Nepal; 2 Nepal Institute of Development Studies, Kathmandu, Nepal

**Keywords:** *postpartum depression*, *prevalence*, *tertiary health care*

## Abstract

**Introduction:**

Postpartum Depression is an important public health problem in developing country like Nepal.Although prevalence of postpartum depression is high in our setting, it is most neglected area of mental health. These have negative consequences not only to mother but also to infant. Data related to postpartum depression in Nepal are limited, research in this particular field will contribute in knowing the gravity of the situation and helps to formulate the factor association to upcoming researchers. This research is done in order to find out the prevalence of postpartum depression.

**Methods:**

A descriptive cross-sectional study was carried out in Paropakar Maternity and Women's Hospital, among total 348 postnatal mothers who were selected through convenient sampling technique. Validated Nepalese version of Edinburg Postnatal Depression Scale was used to screen depressive symptoms. Data was collected after receiving ethical approval letter. Data entry was done using SPSS version 20.

**Results:**

Out of total mothers, the prevalence of Postpartum Depression (PPD) was seen among 51 (14.7%) mothers.

**Conclusions:**

Postpartum Depression being a common yet neglected area of maternal health in Nepal, should be detected in early stage. As the study showed that about one sixth of mothers had postpartum depression, more focus should be given to maternal mental health.

## INTRODUCTION

Postpartum Depression is one of the mental health problems that is a complex mix of physical, emotional, as well as behavioral changes occurring in women after giving birth. It can occur within 4 weeks after delivery, or even 3-12 months. According to the Diagnostic and Statistical Manual of Mental Disorders, fourth edition (DSM-IV), an episode of depression is considered to have postpartum onset if it begins within four weeks after delivery.^1^

Maternity care is still poor in Nepal. Just over onethird of women nation-wide have access to skilled birth attendants.^2^ Postpartum depression (PPD) is a neglected area of maternal health care in developing countries like Nepal. However, it is important to identify and treat postpartum depression because it can have grave consequences for both the mother and her children.^3^

The main aim of this study was to determine the prevalence of postpartum depression among postpartum mothers attending a child immunization clinic at a maternity hospital in Kathmandu, Nepal.

## METHODS

This descriptive cross-sectional was conducted in Paropakar Maternity and Women's Hospital, Kathmandu. The study population was mothers with 6 to 10 weeks postpartum, who attended an immunization clinic. Data collection period was one month from June 2019 to July 2019.

The ethical approval of the study was obtained from the Institutional Review Board of Paropakar Maternity and Women's Hospital (PMWH), Thapathali (Ref No. 59-11-2171). Letter of approval was taken from Paropakar Maternity and Women's Hospital (PMWH) for data collection. Written informed consent from the participants was taken before starting the interview.

The inclusion criteria of the study were mothers at 6-10 weeks of postpartum period who did not have any psychiatric problem, attending immunization clinic who provided consent to be a part of this research. Similarly, women who were not well due to perinatal medical complications, who had pre-existing chronic physical and mental illness were excluded. The sample of 348 mothers were selected by using convenient sampling.

Prevalence i.e. 29% was taken from a study conducted in Nepal.3 Sample size was calculated by using the formula given below:

Sample size (n) = Z^2^ × p × q/ e^2^

                         = (1.96)^2^× 0.29 × 0.71/(0.05)^2^

                         = 0.7909/0.0025

                         = 316

Where,

n= sample size
p= prevalence= 29% =0.29
q= 1-p= 1-0.29= 0.71
e= margin of error= 5%= 0.5
Z=1.96 at 95% confidence interval

Hence, taking non-response rate of 10%, the sample size for the study was calculated and found to be 348. The tool used for data collection in the study was Semi structured questionnaire with Validated Nepalese version Edinburg Postnatal Depression Scale. Edinburg Postnatal Depression Scale was used to calculate prevalence of Postpartum Depression with cut-off value of ≥12 which screened for depressive symptoms. Semi structured questionnaire consisted of Sociodemographic profile. Statistical analysis was done with SPSS 23 version.

## RESULTS

Among 348 respondents, the prevalence of postpartum depression was 51(14.7%) (EPDS score > 12) (Table 1).

**Table 1:** Prevalence of Postpartum Depression (n=348).

Postpartum Mothers with Depressive Symptoms (EPDS > 12)	n(%)
Yes	51(14.7)
No	297(85.3)

Among 348 respondents interviewed, the mean age was 26 (SD: 4.96) and almost half of them 176 (50.6%) were 16-25 years of age. The majority of mothers were Hindus 266 (76.4%), housewives 294 (84.5%) belonging to non-Dalit ethnicity 317 (91.1%) and literate 294 (84.5%). Almost 298 (85%) of households had an average family income of less than 50,000 Nepalese Rupees per month. All of the respondents were married in which about 199 (57.2%) had a love marriage and most of them 216 (62.1%) were from nuclear families. Majority of the respondents gave birth to the male child 183 (52.6%) (Table 2).

**Table 2:** Socio-demographic factors of postpartum mothers (n=348).

Variables	
n(%)	Age
16-25	176 (50.6)
26-35	161 (46.3)
36-45	11 (2.9)
Religion	
Hindu	266 (76.4)
Non-Hindu	82 (23.6)
Ethnicity	
Dalit	31 (8.9)
Non-Dalit	317 (91.1)
Educational status	
Illiterate	54 (15.5)
Literate	294 (84.5)
Occupation	
Employed	54 (15.5)
Housewife	94 (84.5)
Marital Status	
Married	348 (100)
Monthlyincome(NRs)	
<50000	298 (85.6)
>50000	50 (14.4)
Family type	
Joint	132 (37.9)
Nuclear	216 (62.1)
Marriage type	
Love marriage	199 (57.2)
Arranged marriage	149 (42.8)
Sex of baby	
Male	183 (52.6)
Female	165 (47.4)

Among the subgroup, almost 40% of mothers 36-45 years of age had depressive symptoms. Depression among mothers who were literate, non-Hindu and Dalit were 15.3%, 17.1% and 25.8% respectively. Likewise, 16.4% of the mothers whose households had an average family income of less than 50,000 Nepalese Rupees per month and belonged to joint family (13%) were more likely to develop depressive symptoms. 17.6% of the mothers who had love marriage and gave birth to a female child (15.2%) developed depressive symptoms.

## DISCUSSION

The prevalence of Postpartum Depression in this study was found to be 14.7% which was much lower than the level reported in the previous study conducted in Nepal; that reported the prevalence of 29%.^3^ Also, a recent study conducted in PMWH showed the prevalence slightly higher; 17.1% than this study.^4^

The prevalence of PPD amongst Nepalese women assessed by the EPDS screening test (14.7%) was comparatively lower to the results obtained using this test in many other developed countries, including Norway 10.1%^5^ Sweden 12.5%,^6^ and Canada 8.46%.^7^ However, it is higher in developing countries like India 22%,^8^ Pakistan 28.8%,^9^ Indonesia 22.35%,^10^ Argentina 18.6%,^11^ Bangladesh 22%.^12^ The results clarified that PPD was most common in developing countries and especially high in Asian countries. This might be because women in developed countries have access to different quality health services like family planning, reproductive health programs, ANC and PNC services, safe delivery services, promotion of maternal nutrition, infant care and so on.

Another study conducted in Nepal showed a prevalence of PPD 30% which was much higher than this study. This might be due to a higher cut off point of EPDS score (>12) compared to the previous study.13 Findings were consistent with that of Kunwar D who reported that depression was most common among postpartum mothers who were housewives, belonged to Dalit ethnicity.^3^

In the present study, postpartum depression was more in older women (36-45 years) which might be because older women might have difficulty to cope up with the outcomes of pregnancy as compared to mothers of younger age. Employed postpartum mothers might have an extra burden of work as compared to housewives which might be a factor to cause depression after childbirth.

Our study has some limitations. Firstly, the EPDS used is only a symptomatic assessment of depression and is not a clinical diagnosis. Also, the immunization clinic was selected as our setting for data collection so, findings cannot be generalized in a community setting.

## CONCLUSIONS

Postpartum Depression a common yet neglected area of maternal health in Nepal. If detected in early stage, help can be provided and many unnecessary inconveniences could be avoided. This study shows that about one sixth of mothers had postpartum depression. Maternal mental health should also be given equal priority. It can be reliably detected by using the EPDS screening test. These results may have implications for the planning of mental health resources for women in other developing countries. Policies can be formulated to encourage postpartum women to obtain adequate rest during pregnancy, support women with poor partner relationship, reduce marital dissatisfaction, help women adjust with stressful life events, and prevent and manage abortion appropriately. These policies may reduce harmful consequences of postpartum depressive symptoms for women, newborn and their family.
